# Trousseau syndrome-induced cerebral infarction: Two case reports

**DOI:** 10.1097/MD.0000000000040937

**Published:** 2024-12-13

**Authors:** Yongzhen Chen, Qiuxia Wan, Shanshan Li, Bo Liu

**Affiliations:** a Department of Neurology, Shenzhen Longhua District Central Hospital, Shenzhen, Guangdong, P. R. China; b Department of Hematology, Shenzhen Longhua District Central Hospital, Shenzhen, Guangdong, P. R. China.

**Keywords:** case report, cerebral infarction, 3-territory sign, Trousseau syndrome, tumor

## Abstract

**Rationale:**

As a paraneoplastic syndrome, Trousseau syndrome (TS) is a collective term for various thromboembolic events caused by clotting and fibrinolytic abnormalities in patients with tumors, clinically manifesting as venous and arterial thromboembolism, as well as disseminated intravascular coagulation (DIC). The incidence rate of arterial thrombosis in patients with TS is 2% to 5%.

**Patient concerns:**

This article reports 2 patients with TS-induced cerebral infarction. One patient had been definitively diagnosed with cervical adenosquamous carcinoma (stage IVB) accompanied by metastases to the liver and scapulae on May 18, 2020, and was treated with surgery and chemoradiotherapy. The other patient had received laparoscopic radical surgery for distal gastric cancer on March 5, 2018, and had undergone postoperative chemotherapy.

**Diagnoses:**

Both current illnesses had a stroke-like onset, and cranial magnetic resonance imaging (MRI) results were in line with cerebral infarction changes. Hematological examination of both patients revealed an obviously increased D-dimer level. The results for Case 2 also indicated deep-venous thrombosis of the right lower extremity. The 2 patients were finally diagnosed with TS, which was ameliorated after anticoagulant (low-molecular-weight heparin [LMWH]) treatment.

**Lessons:**

Here, the clinical characteristics and treatment of these 2 TS patients are analyzed and the relevant literature is reviewed to improve understanding, diagnosis, and treatment of the disease. Cerebral infarction is the initial symptom in some patients with malignancies. For unexplained multiple cerebral infarctions, we should screen for occult malignancies to facilitate early diagnosis and treatment, as early and accurate identification of the cause of the disease may improve prognosis.

## 1. Introduction

As a paraneoplastic syndrome, Trousseau syndrome (TS) is a collective term for various thromboembolic events caused by clotting and fibrinolytic abnormalities in patients with tumors, clinically manifesting as venous and arterial thromboembolism, as well as disseminated intravascular coagulation (DIC).^[1]^ The disease was first reported by Trousseau in 1865, who suggested that patients with gastric cancer were prone to experience venous thrombosis.^[2]^

Cerebral infarction is the most common outcome of arterial embolism, and magnetic resonance imaging (MRI) shows multiple lesions scattered in more than 2 cerebral regions, often involving both bilateral anterior and posterior circulation territories. Multifocal and multiregional acute cerebral infarctions are usually caused by cardiogenic embolism in clinical practice, but the significance of investigating TS-induced acute cerebral infarctions is not fully understood.

In this study, the clinical and imaging characteristics of 2 patients with TS-induced acute cerebral infarction were retrospectively analyzed to improve clinicians’ understanding of TS.

## 2. Case presentation

### 2.1. Chief complaints

Case 1: A 67-year-old female was admitted to the hospital on April 22, 2023, due to “inability to speak and limb weakness for 2 days.”

Case 2: A 48-year-old female was admitted to the hospital on June 1, 2023, due to “cough for 1 month and expressive language disorder and memory decline for 4 days.”

### 2.2. History of present illness

Case 1: The patient visited the hospital with sudden onset of persistent inability to speak and limb weakness without obvious predisposing factors.

Case 2: The patient had a recurrent cough without obvious predisposing factors a month prior, without sputum, chest tightness, shortness of breath, or other discomforts. Four days before admission, she suddenly developed expressive language disorder and memory decline, accompanied by slow responses. The above symptoms progressively worsened.

### 2.3. History of past illness

Case 1: The patient had been definitively diagnosed with cervical adenosquamous carcinoma (stage IVB) accompanied by metastases to the liver and scapulae on May 18, 2020, for which she had been treated with surgery and chemoradiotherapy.

Case 2: The patient had undergone laparoscopic radical surgery for distal gastric cancer at Xiangya Hospital, Central South University, on March 5, 2018. Pathological diagnosis at that time suggested poorly differentiated gastric adenocarcinoma, partly signet-ring cell carcinoma (ulcerative type, with a maximum diameter of 3 cm), invading the serosa. Metastasis was found in the following groups of lymph nodes sent for pathological examination: 1/5 in group 4, 5/7 in group 6, 1/5 in group 7, 1/1 in group 11, and 1/1 in group 12. Immunohistochemical staining indicated HER-2 (0), CK-Pan (+), CD31 (−), D2-40 (−), EVG (−), and PAS + lining stain (+). She received 8 sessions of postoperative chemotherapy. Annual endoscopic reexamination showed no tumor recurrence. The patient stated a history of moderate iron-deficiency anemia in 2021, which was cured.

### 2.4. Personal and family history

Both patients denied any family history of tumors or strokes, and both had healthy, nonconsanguineous parents.

### 2.5. Physical examination

Case 1: Physical examination revealed a body temperature of 36.2 °C, pulse of 79 beats/min, respiration of 20 breaths/min, and blood pressure of 122/86 mm Hg. Her weight was 55 kg, and her height was 156 cm. The patient had a conscious mind, motor aphasia, grade 4 muscle strength in all 4 limbs, and bilateral Babinski sign (+). No significant abnormalities were observed in any other systems. Her National Institute of Health Stroke Scale (NIHSS) score was 7 points.

Case 2: Physical examination revealed a body temperature of 36.5 °C, pulse of 84 beats/min, respiration of 20 breaths/min, and blood pressure of 113/73 mm Hg. Her body weight was 52 kg, and her height was 158 cm. The patient had a conscious mind, incomplete motor aphasia, memory decline, and slow responses, as well as coarse breath sounds in both lungs. No significant abnormalities were observed in any other systems. Her NIHSS score was 1 point.

### 2.6. Laboratory examinations

Case 1: Laboratory results showed a white blood cell count of 5.76 × 10^9^/L, hemoglobin 94 g/L ↓, platelets 103 × 10^9^/L ↓, thrombin time 15.2 seconds, prothrombin time 11.4 seconds, activated partial thromboplastin time 21.5 seconds ↓, fibrinogen quantification 3.90 g/L, international normalized ratio 0.99, D-dimer 14.63 μg/mL ↑, triglycerides 1.83 mmol/L, low-density lipoprotein cholesterol 1.79 mmol/L, and erythrocyte sedimentation rate 59.00 mm/h ↑.

Case 2: Laboratory results showed a white blood cell count of 5.28 × 10^9^/L, hemoglobin 131 g/L, platelets 195 × 10^9^/L, thrombin time 15.9 seconds, prothrombin time 12.8 seconds, activated partial thromboplastin time 28.0 seconds, fibrinogen quantification 3.41 g/L, international standardized ratio 1.12, D-dimer 3.81 μg/mL ↑, triglycerides 1.37 mmol/L, low-density lipoprotein cholesterol 2.42 mmol/L, erythrocyte sedimentation rate 21.00 mm/h ↑, carcinoembryonic antigen 5.59 ng/mL ↑, and high-sensitivity C-reactive protein 5.92 mg/L.

### 2.7. Imaging examinations

**Figure 1.**
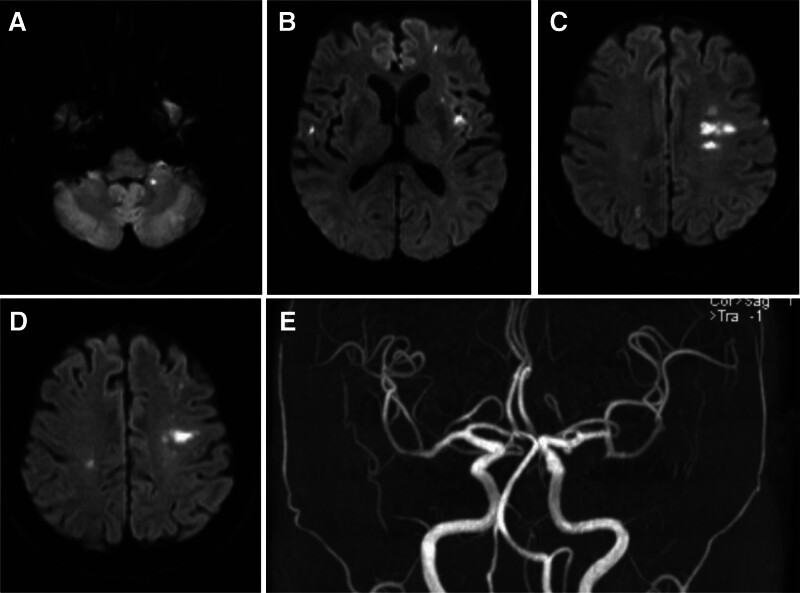
Images of Case 1. (A) MRI shows acute cerebral infarctions in the left cerebellar hemisphere. (B) MRI shows acute cerebral infarctions in the left frontotemporal lobe, basal ganglia region, and right temporal lobe. (C) MRI shows acute cerebral infarctions in the left frontoparietal lobe and right parieto-occipital lobe. (D) MRI shows acute cerebral infarctions in the bilateral parietal lobes. (E) There were no obvious abnormalities on time-of-flight MRA scanning. MRA = magnetic resonance angiography, MRI = magnetic resonance imaging.

Case 1: Cranial MRI + magnetic resonance angiography (MRA): multiple scattered acute cerebral infarctions in the left frontotemporal lobe, basal ganglia region, left cerebellar hemisphere, and right frontal, parietal, temporal, and occipital lobes (Fig. 1A–D). There were no obvious abnormalities on time-of-flight MRA scanning (Fig. 1E). Arterial vascular ultrasonography: normal internal diameters of visual segments in bilateral common carotid arteries, internal carotid arteries and vertebral arteries; smooth arterial blood flow; and no abnormalities in the spectral morphology and parameters. Liver ultrasonography: a solid space-occupying lesion in the liver, which was considered a metastatic tumor. Electrocardiogram showed no abnormalities. Cardiac ultrasonography: ejection fraction (EF) 65%, mild mitral regurgitation, and nearly normal systolic function of the left ventricle.

**Figure 2.**
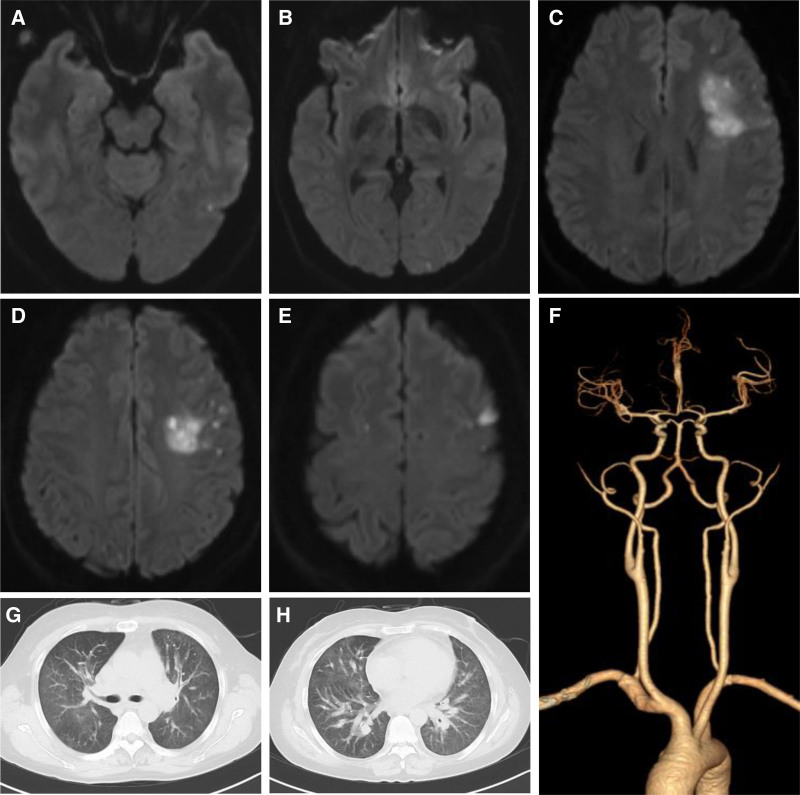
Images of Case 2. (A) MRI shows acute cerebral infarctions in the left temporal lobe. (B) MRI shows acute cerebral infarctions in the left occipital lobe. (C) MRI shows acute cerebral infarctions in the left frontoparietal lobe and bilateral occipital lobes. (D) MRI shows acute cerebral infarctions in the left frontoparietal lobe. (E) MRI shows acute cerebral infarctions in the bilateral parietal lobes. (F) Head and neck CTA showed no obvious abnormalities. (G) Chest computed tomography (CT): pulmonary parenchyma, tracheal wall edema and thickening, and mediastinal edema. (H) Chest CT: pulmonary parenchyma, tracheal wall edema and thickening, and mediastinal edema. CTA = computed tomography angiography, MRI = magnetic resonance imaging.

Case 2: Cranial MR: acute cerebral infarctions in the left frontal, insular, temporal and occipital lobes and right frontal lobe (Fig. 2A–E). Head and neck computed tomography angiography showed no obvious abnormalities (Fig. 2F). Electronic gastroscopy: inflammation of the gastric relict after Billroth I subtotal gastrectomy. Electrocardiogram: sinus rhythm; extensive anterior and inferior wall ST-T abnormalities. Cardiac ultrasonography: EF 68%, mild mitral regurgitation, and normal systolic and diastolic functions of the left ventricle. The foaming test was negative. Vascular ultrasonography of lower extremities: intermuscular vein thrombosis of the right lower leg. Chest computed tomography (CT) showed pulmonary parenchyma, tracheal wall edema and thickening, and mediastinal edema (Fig. 2G and H). Pulmonary function tests: extremely severe mixed ventilation dysfunction and a negative result on the bronchial dilation test. Measurement of pulmonary diffusion function: abnormal diffusion capacity and moderate dysfunction of gas exchange in the lungs. Bronchial mucosal biopsy: mucosal edema, infiltration of inflammatory cells (mainly lymphocytes), acid-fast staining (−), and hexamine silver staining (−). Positron-emission tomography (PET)/CT: no signs of tumor recurrence in the operative area after surgery for gastric carcinoma and no signs of hypermetabolic lymph node metastasis in the abdominal or pelvic cavities; diffusely distributed lesions in both lungs, with mildly to moderately elevated metabolism, considered lymphangitis carcinomatosa of both lungs; focally elevated metabolism in the left lobe of the liver, basically the same delayed scan metabolism as before, and slightly lower density on CT images, suggesting metastases to the liver; and inflammatory hyperplasia of the left cervical lymph nodes.

### 2.8. Final diagnosis

Case 1: TS, cerebral infarction, cervical adenosquamous carcinoma (stage IVB) accompanied by metastases to the liver and scapulae, and posthysterectomy status of cervical cancer.

Case 2: TS, cerebral infarction, lymphangitis carcinomatosa of both lungs, metastatic tumors of the liver, and postoperative status of gastric cancer.

### 2.9. Treatment

Case 1: During hospitalization, the patient’s condition was assessed daily, and anticoagulant (low-molecular-weight heparin [LMWH]) therapy and rehabilitation therapy were given.

Case 2: During hospitalization, the patient’s condition was evaluated every day, anticoagulant (LMWH) therapy and rehabilitation therapy were given, and doxofylline, salbutamol, and glucocorticoids were administered to ameliorate her respiratory symptoms.

### 2.10. Outcome and follow-up

Case 1: The patient’s motor aphasia and limb weakness were relieved, and the NIHSS score decreased to 2 points. She was referred to a cancer hospital to continue chemotherapy. The patient who was followed up for 1 years had stable conditions and was able to take care of himself, with an mRS score of 1.

Case 2: The patient’s expressive language disorder and memory decline were ameliorated, and her NIHSS score decreased to 0 points. However, the patient’s cough worsened, and she gradually developed discomfort such as chest tightness and dyspnea and was referred to a cancer hospital to continue chemotherapy. The patient died of severe pneumonia 3 months later.

## 3. Discussion

Cancer-associated thrombosis is characterized by deep-venous thrombosis (DVT), pulmonary embolism, chronic DIC complicated with nonbacterial thrombotic endocarditis, and arterial thrombosis in the clinic. It is estimated that the incidence rate of arterial thrombosis is 2% to 5%, accounting for 10% to 30% of all thrombotic complications, and TS is the second-leading cause of cancer-related death, only after cancers.^[3]^ In 1865, Armand Trousseau, a French physician, reported a patient with gastrointestinal discomfort accompanied by migrating thrombophlebitis who was diagnosed with gastric cancer 6 months later.^[4]^ Over the decades, there have been more reports on the association of tumors and thrombosis, showing a close relationship. In diagnosis of TS, a hypercoagulable state of patients with malignancies, similar to “cancer-associated thrombosis,” is often described.^[1]^ The cause of malignancy-associated cerebral infarction remains unclear and may be attributed to the following factors. Reduction of anticoagulant substances^[5]^: vascular endothelial cells are destroyed by malignant tumors, resulting in downregulation of factors that antagonize thrombin or coagulation factors, such as thrombomodulin (TM) and antithrombin III; tumor cells can trigger mononuclear macrophages to secrete TNF, IL-1, and tissue factor pathway inhibitor (TFPI) with downregulated oxygen radicals, leading to downregulation of protein C system anticoagulation; massive anticoagulant substances are consumed. Activation of the coagulation system: damaged endothelial cells are prone to mingling with tumor cells, thus activating the cell system in the body, which produces cytokines to activate the coagulation system^[6]^; Inhibited fibrinolytic system^[7]^: tumor cells secrete fibrinogen activator inhibitor-1 to repress tissue-type and urokinase-type plasminogen activators, impeding fibrinolytic function; large amounts of tissue-type plasminogen are released by damaged vascular endothelial cells, weakening fibrinolytic function; Thrombosis due to administration of chemotherapeutic drugs: chemotherapeutic drugs may lead to vascular endothelial damage, initiating the coagulation mechanism; chemotherapeutic drugs can induce platelet activation and aggregation; chemotherapeutic drugs can reduce levels of protein S, protein C and other anticoagulant substances; and Radiotherapy: the blood vessels around lesions may be damaged during radiotherapy, and damaged blood vessels can activate corresponding inflammatory responses to promote coagulation, giving rise to thrombosis.

The incidence of lung cancer is highest among TS patients, occurring in approximately 73.3%.^[8]^ Common pathophysiological changes include local hypercoagulability of the blood. Adenocarcinoma is the most common primary tumor pathologic type, which may be related to the fact that mucin secreted by adenocarcinoma leads to microthrombosis. In this study, the primary tumor in Case 1 and Case 2 was cervical adenosquamous carcinoma and poorly differentiated gastric adenocarcinoma (partial signet-ring cell carcinoma), respectively, consistent with the findings of the above study.

Selvik et al^[9]^ evaluated 1646 patients with ischemic stroke and found that 53% with unexplained ischemic stroke (>75 years old) had active cancers and that elevated D-dimer and decreased hemoglobin are significant for diagnosis of TS. Some studies^[10,11]^ have found that elevated D-dimer (>5.5 or >9 μg/mL) has >93% sensitivity and specificity for predicting thrombosis in patients with occult tumor-associated ischemic stroke and reflects the hypercoagulable state of patients with tumors, but the degree of neurological deficit in acute ischemic stroke (NIHSS score) showed no correlation with D-dimer level. In this study, both patients had decreased hemoglobin and increased D-dimer, confirming the diagnostic value of D-dimer and hemoglobin levels for TS.

MRI, the most important imaging approach for diagnosis of cerebral infarction, has advantages such as high soft-tissue resolution and multidirectional scanning. Nouh et al^[12]^ reported that the 3-territory sign is a forceful imaging marker for ischemic stroke in TS, with high specificity (96.4%). In the present study, diffusion-weighted imaging (DWI) of both patients revealed multiple lesions in many vascular areas, involving bilateral anterior and posterior circulation territories. Both patients had multiple cerebral infarctions (>5), the longest diameter being greater than the upper limit of the lacunar infarction diameter (15 mm). The lesions were mostly located in lobar areas and cerebellar structures but rarely in the brainstem, in keeping with the fact that ischemic stroke in TS patients is characterized by numerous lesions, a large area, and does not often involve deeper brain structures. Therefore, patients with multiple small, bilateral infarctions, especially those with simultaneous involvement of multiple vascular regions of unknown cause, should be carefully examined for possible malignancies. Nevertheless, multiple small infarctions are not a unique feature of cancer patients with stroke, and infarction is also commonly caused by cardiogenic, paradoxical, and aortic cerebral embolisms, which should be identified by clinical examination. In an imaging study comparing acute massive cerebral infarctions induced by TS and cardiogenic and artery-to-artery embolisms, bilateral cerebral embolisms was used as an imaging hallmark of TS.^[13]^

The 2 patients reported herein had stroke-like onset, and their cranial MRI results were consistent with cerebral infarction changes. According to the TOAST classification of cerebral infarction, the patients did not have risk factors such as hypertension, diabetes mellitus, hyperlipidemia, smoking habit, or drinking habit or obvious cerebrovascular stenosis, which suggests an absence of atherosclerosis of large arteries. In addition, the blood pressure of the patients was not low at onset, ruling out the possibility of hypoperfusion, and the imaging results suggested new infarctions in more than 3 blood-supplying areas of bilateral anterior and posterior circulation territories, more likely indicating embolism rather than small-artery occlusion. On the other hand, atrial fibrillation was not detected on the electrocardiogram, no structural abnormalities were found by cardiac ultrasonography, and no embolic signals were observed via transcranial Doppler ultrasound monitoring, making cardiogenic embolism unlikely. Hematological examination of the 2 patients showed obviously increased D-dimer levels. They both had a history of tumors accompanied by metastases and a history of chemotherapy drug administration. DVT of the right lower extremity was found in Case 2. The 2 patients were finally diagnosed with TS, which we considered related to multiple in situ thromboses in cerebral vessels caused by the hypercoagulable state of their malignancies.

Currently, anticoagulation is the preferred treatment for TS, and it is performed along with treatment of the primary tumor. LMWH is the first choice for treatment and prevention of cancer-associated thromboembolic events and should be administered for at least 3 to 6 months.^[14]^ LMWH can relieve the hypercoagulable state in patients with tumor-related stroke and treat venous thrombotic diseases. Donati^[15]^ suggested that LMWH may be able to delay the metastasis and growth of early tumors by transferring cancer cells to the spleen or liver, where these cells are destroyed by the reticuloendothelial system. One randomized controlled study comparing direct oral anticoagulant (DOAC) with LMWH in cancer patients did not recommend using DOAC for treatment of cancer-associated venous thromboembolism,^[14]^ though warfarin may be ineffective for unknown reasons. In studies of ultraearly ischemic stroke in TS patients,^[16,17]^ after excluding other contraindications to thrombolysis, such as intracranial metastatic tumors or hemorrhage, intravenous thrombolysis did not increase the risk of hemorrhagic complications in patients with ischemic stroke who met the indications for thrombolysis, and the clinical symptoms of such patients were significantly relieved after intravenous thrombolysis. Some patients have benefited from endovascular intervention.^[18]^

A previous study^[19]^ demonstrated that most patients with TS-induced ischemic infarction have poor prognosis, a high recurrence rate, and a mortality rate of 25% to 30%. This may be attributed to a combination of poor baseline status, chronic hypercoagulable state, and tumor-related comorbidities.

## 4. Conclusions

TS should be highly suspected in the case of acute ischemic stroke manifesting as elevated plasma D-dimer, decreased hemoglobin, elevated C-reactive protein, and multiple lesions involving multiple blood-supplying areas (especially bilateral anterior and posterior circulation territories), as observed on DWI MRI for patients with advanced age. Cerebral infarction may be the initial symptom in some patients with malignancies. For this reason, effort should be made to screen for occult malignancies to facilitate early diagnosis and treatment, as early and accurate identification of the cause of the disease may improve prognosis.

## Author contributions

**Conceptualization:** Bo Liu, Yongzhen Chen.

**Data curation:** Bo Liu, Yongzhen Chen, Qiuxia Wan, Shanshan Li.

**Formal analysis:** Bo Liu, Yongzhen Chen, Shanshan Li.

**Funding acquisition:** Bo Liu, Yongzhen Chen.

**Investigation:** Bo Liu, Yongzhen Chen, Qiuxia Wan, Shanshan Li.

**Methodology:** Bo Liu, Yongzhen Chen.

**Project administration:** Bo Liu, Yongzhen Chen.

**Resources:** Bo Liu, Yongzhen Chen, Qiuxia Wan, Shanshan Li.

**Software:** Bo Liu, Yongzhen Chen.

**Supervision:** Bo Liu, Yongzhen Chen.

**Validation:** Bo Liu, Yongzhen Chen.

**Visualization:** Bo Liu, Yongzhen Chen.

**Writing – review & editing:** Bo Liu.

**Writing – original draft:** Yongzhen Chen.

## References

[1] Ikushima S Ono R Fukuda K Sakayori M Awano N Kondo K. Trousseau’s syndrome: cancer-associated thrombosis. Jpn J Clin Oncol. 2016;46:204–8. 26546690 10.1093/jjco/hyv165

[2] Trousseau A. Clinique Medicale de l’Hotel Dieu de Paris. Paris: Balliere.1865;3:654–6.

[3] Khorana AA. Venous thromboembolism and prognosis in cancer. Thromb Res. 2010;125:490–3. 20097409 10.1016/j.thromres.2009.12.023 PMC2878879

[4] Trousseau A. Plegmasia alba dolens. Lectures on clinical medicine. Paris.1865;5:281–332.

[5] Dammacco F Vacca A Procaccio P Ria R Marech I Racanelli V. Cancer-related coagulopathy (Trousseau’s syndrome): review of the literature and experience of a single center of internal medicine. Clin Exp Med. 2013;13:85–97. 23456539 10.1007/s10238-013-0230-0

[6] Repetto O De RV. Coagulation and fibrinolysis in gastric cancer. Ann N Y Acad Sci. 2017;1404:2748. 10.1111/nyas.13454 28833193

[7] Varki A. Trousseau’s syndrome: multiple definitions and multiple mechanisms. Blood. 2007;110:1723–9. 17496204 10.1182/blood-2006-10-053736 PMC1976377

[8] Ling Y Li Y Zhang X Dong L Wang J. Clinical features of Trousseau’s syndrome with multiple acute ischemic strokes. Neurol Sci. 2022;43:2405–11. 34564800 10.1007/s10072-021-05619-y

[9] Selvik HA Bjerkreim AT Thomassen L Waje-Andreassen U Naess H Kvistad CE. When to screen ischaemic stroke patients for cancer. Cerebrovasc Dis. 2018;45:42–7. 29402826 10.1159/000484668

[10] Guo YJ Chang MH Chen PL Lee YS Chang YC Liao YC. Predictive value of plasma (D)-dimer levels for cancer-related stroke: a 3-year retrospective study. J Stroke Cerebrovasc Dis. 2014;23:e249–54. 24295603 10.1016/j.jstrokecerebrovasdis.2013.10.022

[11] Ito S Kikuchi K Ueda A. Changes in serial D-dimer levels predict the prognoses of Trousseau’s syndrome patients. Front Neurol. 2018;9:528. 30018592 10.3389/fneur.2018.00528 PMC6037767

[12] Nouh AM Staff I Finelli PF. Three territory sign: an MRI marker of malignancy-related ischemic stroke (Trousseau syndrome). Neurol Clin Pract. 2019;9:124–8. 31041126 10.1212/CPJ.0000000000000603 PMC6461422

[13] Umemura T Yamamoto J Akiba D Nishizawa S. Bilateral cerebral embolism as a characteristic feature of patients with Trousseau syndrome. J Clin Neurosci. 2017;42:155–9. 28431956 10.1016/j.jocn.2017.04.014

[14] Franco-Moreno A Cabezón-Gutiérrez L Palka-Kotlowsa M Villamayor-Delgado M García-Navarro M. Evaluation of direct oral anticoagulants for the treatment of cancer-associated thrombosis: an update. J Thromb Thrombolysis. 2019;47:409–19. 30467760 10.1007/s11239-018-1783-2

[15] Donati MB. Thrombosis and cancer: Trousseau syndrome revisited. Best Pract Res Clin Haematol. 2009;22:3–8. 19285268 10.1016/j.beha.2009.01.005

[16] Vedovati MC Germini F Agnelli G Becattini C. Direct oral anticoagulants in patients with VTE and cancer a systematic review and meta-analysis. Chest. 2015;147:475–83. 25211264 10.1378/chest.14-0402

[17] Sakai S Tsurusaki Y Morita T. Multiple thrombectomies for cerebral and coronary artery occlusion in Trousseau syndrome. J Neuroendovasc Ther. 2022;16:116–22. 37502648 10.5797/jnet.cr.2021-0027 PMC10370965

[18] Sakuta K Mukai T Fujii A Makita K Yaguchi H. Endovascular therapy for concurrent cardio-cerebral infarction in a patient with Trousseau syndrome. Front Neurol. 2019;10:965. 31555206 10.3389/fneur.2019.00965 PMC6742686

[19] Zhen C Wang Y Wang H Li D Wang X. Multiple cerebral infarction linked to underlying cancer: a review of Trousseau syndrome-related cerebral infarction. Br J Hosp Med (Lond). 2021;82:1–7. 10.12968/hmed.2020.0696 34076507

